# Supplementary material for: MetaRibo-Seq measures translation in microbiomes
Source: Nat Commun. 2020 Jun 29;11:3268. doi: 10.1038/s41467-020-17081-z (PMC7324362; doi:10.1038/s41467-020-17081-z)
Supplement: Supplementary file 10 — Supplementary Data 7 [file 41467_2020_17081_MOESM10_ESM.zip › File2/Confidence_High_Taxonomy/402819_out.krona.html]

Javascript must be enabled to view this page.

members
magnitude
magnitudeUnassigned
count
unassigned
taxon
rank

402819\_out

284

2
superkingdom
284

phylum
280
1239

1879010

SRS971276\_contig\_number\_31467
species
1

33
species

SRS013521\_contig\_number\_10095SRS015431\_contig\_number\_28861SRS015579\_contig\_number\_16079SRS016203\_contig\_number\_contig-100\_6363.66703SRS019445\_contig\_number\_20377SRS024435\_contig\_number\_contig-100\_2418.242084SRS048870\_contig\_number\_18456SRS049773\_contig\_number\_22365SRS049959\_contig\_number\_43150SRS052697\_contig\_number\_14203SRS053356\_contig\_number\_12331SRS053398\_contig\_number\_contig-100\_2264.139444SRS055017\_contig\_number\_contig-100\_364.216377SRS075773\_contig\_number\_24658SRS075984\_contig\_number\_20964SRS078176\_contig\_number\_contig-100\_1071.135085SRS1041032\_contig\_number\_8338SRS1041039\_contig\_number\_24064SRS1041092\_contig\_number\_13001SRS1041129\_contig\_number\_12931SRS1041133\_contig\_number\_5078SRS1041141\_contig\_number\_12154SRS1041145\_contig\_number\_33993SRS104400\_contig\_number\_45572SRS142542\_contig\_number\_21597SRS143417\_contig\_number\_33084SRS143466\_contig\_number\_23937SRS144506\_contig\_number\_24056SRS144537\_contig\_number\_1322SRS146813\_contig\_number\_contig-100\_8028.115684SRS148817\_contig\_number\_4064SRS149325\_contig\_number\_2456SRS150029\_contig\_number\_14468
1263003


SRS022524\_contig\_number\_14208
1
species
1263002

186801
class
245

2508939
1
order

family
1
2508947

1
genus
2508948

1
species

SRS011084\_contig\_number\_31802
2508949

244
order
186802

31979
1
family

genus
1
580596

2292294

SRS143181\_contig\_number\_11459
species
1

species
1

SRS076929\_contig\_number\_24029
2109688


SRS011239\_contig\_number\_25021SRS011302\_contig\_number\_contig-100\_2180.161257SRS012969\_contig\_number\_21331SRS013098\_contig\_number\_2521SRS013965\_contig\_number\_28891SRS015694\_contig\_number\_13011SRS016335\_contig\_number\_32684SRS018351\_contig\_number\_3834SRS018427\_contig\_number\_13463SRS019161\_contig\_number\_18928SRS019496\_contig\_number\_10120SRS020869\_contig\_number\_contig-100\_2236.185134SRS021484\_contig\_number\_31472SRS021948\_contig\_number\_contig-100\_6718.156108SRS022071\_contig\_number\_6050SRS023526\_contig\_number\_contig-100\_572.84423SRS023914\_contig\_number\_16333SRS042628\_contig\_number\_contig-100\_2806.108803SRS042966\_contig\_number\_1300SRS045645\_contig\_number\_31376SRS045826\_contig\_number\_20343SRS047433\_contig\_number\_contig-100\_10935.83830SRS050752\_contig\_number\_11671SRS050925\_contig\_number\_32152SRS054352\_contig\_number\_3997SRS054905\_contig\_number\_22506SRS056273\_contig\_number\_18083SRS058070\_contig\_number\_contig-100\_1189.189742SRS062654\_contig\_number\_5777SRS063190\_contig\_number\_9699SRS064276\_contig\_number\_34166SRS064757\_contig\_number\_15855SRS065504\_contig\_number\_24371SRS075078\_contig\_number\_18811SRS075341\_contig\_number\_24494SRS075821\_contig\_number\_18082SRS077024\_contig\_number\_10398SRS077641\_contig\_number\_contig-100\_3050.133126SRS077849\_contig\_number\_30870SRS078242\_contig\_number\_contig-100\_627.200229SRS078419\_contig\_number\_contig-100\_3297.193615SRS098571\_contig\_number\_48215SRS098644\_contig\_number\_12510SRS101376\_contig\_number\_contig-100\_10928.115162SRS1041139\_contig\_number\_4620SRS1041140\_contig\_number\_19099SRS1041157\_contig\_number\_3506SRS104693\_contig\_number\_contig-100\_6473.54244SRS104912\_contig\_number\_contig-100\_2220.122143SRS140513\_contig\_number\_contig-100\_3180.41745SRS142503\_contig\_number\_54842SRS143523\_contig\_number\_18494SRS143895\_contig\_number\_17286SRS144183\_contig\_number\_32573SRS146812\_contig\_number\_22854SRS147271\_contig\_number\_contig-100\_2842.230427SRS147557\_contig\_number\_24726SRS147614\_contig\_number\_780SRS148196\_contig\_number\_35322SRS148253\_contig\_number\_10535SRS892882\_contig\_number\_12021SRS893172\_contig\_number\_9486SRS893358\_contig\_number\_13420SRS893366\_contig\_number\_7906SRS893378\_contig\_number\_4287SRS893383\_contig\_number\_24952SRS971275\_contig\_number\_14665
67
species
172733


SRS077392\_contig\_number\_15514SRS148721\_contig\_number\_20098
species
2
1950935

13
family

SRS018836\_contig\_number\_contig-100\_5475.132284
541000
1

species
2

SRS019496\_contig\_number\_1107SRS046502\_contig\_number\_10702
2292270


SRS012273\_contig\_number\_27368SRS045739\_contig\_number\_11168SRS1041137\_contig\_number\_22682
3
species
1898205

2292269
1
species

SRS016018\_contig\_number\_3976

5
genus
216851

2302956
species
1

SRS018984\_contig\_number\_24389


SRS015960\_contig\_number\_11563SRS049995\_contig\_number\_19732SRS056273\_contig\_number\_30915SRS104311\_contig\_number\_7305
4
species
853

292632
genus
1

2053618

SRS893373\_contig\_number\_6275
1
species

216572
160
family

genus
159
459786


SRS011084\_contig\_number\_17911SRS011302\_contig\_number\_contig-100\_4173.151681SRS012849\_contig\_number\_18588SRS013098\_contig\_number\_7806SRS013382\_contig\_number\_1338SRS013521\_contig\_number\_10492SRS013638\_contig\_number\_9134SRS013940\_contig\_number\_18630SRS013951\_contig\_number\_31228SRS014412\_contig\_number\_6039SRS014415\_contig\_number\_1439SRS014459\_contig\_number\_29880SRS014683\_contig\_number\_5193SRS014855\_contig\_number\_8052SRS015065\_contig\_number\_34926SRS015431\_contig\_number\_66486SRS015663\_contig\_number\_14289SRS015663\_contig\_number\_20765SRS015694\_contig\_number\_16941SRS015782\_contig\_number\_contig-100\_3651.232069SRS015890\_contig\_number\_contig-100\_5625.143803SRS016018\_contig\_number\_5544SRS016056\_contig\_number\_contig-100\_9290.46050SRS016203\_contig\_number\_contig-100\_3663.3663SRS016753\_contig\_number\_8575SRS017916\_contig\_number\_17177SRS018351\_contig\_number\_contig-100\_597.80256SRS018541\_contig\_number\_contig-100\_732.84610SRS018623\_contig\_number\_17172SRS018656\_contig\_number\_11607SRS018836\_contig\_number\_7915SRS018888\_contig\_number\_7900SRS019030\_contig\_number\_16043SRS019068\_contig\_number\_62370SRS019397\_contig\_number\_7754SRS019445\_contig\_number\_1891SRS019693\_contig\_number\_7534SRS019808\_contig\_number\_19385SRS019968\_contig\_number\_9545SRS020233\_contig\_number\_5455SRS022137\_contig\_number\_16998SRS022609\_contig\_number\_36574SRS022609\_contig\_number\_45721SRS024075\_contig\_number\_13011SRS024663\_contig\_number\_874SRS043411\_contig\_number\_contig-100\_575.91916SRS043701\_contig\_number\_contig-100\_397.117969SRS043768\_contig\_number\_24646SRS043841\_contig\_number\_contig-100\_482.63937SRS044535\_contig\_number\_1509SRS045826\_contig\_number\_19024SRS047044\_contig\_number\_5313SRS047433\_contig\_number\_21597SRS048164\_contig\_number\_24955SRS049446\_contig\_number\_contig-100\_12936.12937SRS049446\_contig\_number\_contig-100\_866.61559SRS049896\_contig\_number\_24797SRS049995\_contig\_number\_21431SRS050925\_contig\_number\_17265SRS050998\_contig\_number\_12375SRS053356\_contig\_number\_11453SRS053573\_contig\_number\_1543SRS053649\_contig\_number\_19971SRS054059\_contig\_number\_contig-100\_1547.50250SRS054352\_contig\_number\_13124SRS054905\_contig\_number\_contig-100\_4168.125015SRS055017\_contig\_number\_contig-100\_2082.182537SRS055982\_contig\_number\_14236SRS058070\_contig\_number\_845SRS058145\_contig\_number\_contig-100\_12435.12436SRS062654\_contig\_number\_21304SRS062701\_contig\_number\_5267SRS063518\_contig\_number\_35877SRS064276\_contig\_number\_33343SRS064757\_contig\_number\_16772SRS065397\_contig\_number\_6501SRS074586\_contig\_number\_contig-100\_3845.3846SRS074670\_contig\_number\_6242SRS074964\_contig\_number\_1434SRS075078\_contig\_number\_7838SRS075341\_contig\_number\_12356SRS075773\_contig\_number\_36315SRS075878\_contig\_number\_12192SRS075984\_contig\_number\_contig-100\_3783.146625SRS076756\_contig\_number\_480SRS076804\_contig\_number\_contig-100\_1202.132659SRS077127\_contig\_number\_15640SRS077294\_contig\_number\_17788SRS077392\_contig\_number\_1550SRS077392\_contig\_number\_21646SRS077552\_contig\_number\_29135SRS077641\_contig\_number\_23127SRS078176\_contig\_number\_17685SRS097920\_contig\_number\_28960SRS098073\_contig\_number\_318SRS098571\_contig\_number\_37992SRS098644\_contig\_number\_11499SRS098827\_contig\_number\_3991SRS098881\_contig\_number\_26498SRS100907\_contig\_number\_8178SRS101433\_contig\_number\_7593SRS103987\_contig\_number\_33225SRS104036\_contig\_number\_11SRS104084\_contig\_number\_4724SRS1041033\_contig\_number\_16379SRS1041037\_contig\_number\_contig-100\_1014.61875SRS1041092\_contig\_number\_13207SRS1041116\_contig\_number\_2686SRS1041133\_contig\_number\_25682SRS1041134\_contig\_number\_14515SRS1041142\_contig\_number\_7706SRS104327\_contig\_number\_4641SRS104400\_contig\_number\_374SRS104912\_contig\_number\_contig-100\_3669.96471SRS104975\_contig\_number\_22492SRS1055083\_contig\_number\_contig-100\_242.85704SRS1055099\_contig\_number\_7295SRS142503\_contig\_number\_contig-100\_3722.335316SRS142542\_contig\_number\_14973SRS142599\_contig\_number\_27165SRS142890\_contig\_number\_37795SRS143070\_contig\_number\_contig-100\_1145.99709SRS143085\_contig\_number\_2848SRS143148\_contig\_number\_17882SRS143342\_contig\_number\_3059SRS143417\_contig\_number\_2173SRS143523\_contig\_number\_contig-100\_295.107072SRS143523\_contig\_number\_contig-100\_1646.139397SRS143598\_contig\_number\_7031SRS143895\_contig\_number\_44461SRS143991\_contig\_number\_3760SRS144362\_contig\_number\_42109SRS144506\_contig\_number\_36951SRS144603\_contig\_number\_5282SRS145497\_contig\_number\_16061SRS147271\_contig\_number\_contig-100\_1967.210600SRS147346\_contig\_number\_contig-100\_2417.339824SRS147614\_contig\_number\_2125SRS148159\_contig\_number\_6717SRS148253\_contig\_number\_2559SRS148319\_contig\_number\_10218SRS148784\_contig\_number\_27088SRS148817\_contig\_number\_8501SRS149325\_contig\_number\_12491SRS150029\_contig\_number\_1699SRS893172\_contig\_number\_contig-100\_5232.41819SRS893252\_contig\_number\_2116SRS893252\_contig\_number\_7612SRS893292\_contig\_number\_5369SRS893295\_contig\_number\_contig-100\_782.78787SRS893300\_contig\_number\_12658SRS893373\_contig\_number\_1781SRS893378\_contig\_number\_12550SRS971275\_contig\_number\_43410
species
154
1945593

1897011

SRS024435\_contig\_number\_35569SRS077231\_contig\_number\_contig-100\_428.120122SRS078242\_contig\_number\_20751SRS101376\_contig\_number\_1844SRS142923\_contig\_number\_13262
5
species

species
1

SRS043701\_contig\_number\_contig-100\_8111.82977
1952974

phylum
4
976

200643
class
4

171549
4
order

4
family
171550

1929083
1
genus


SRS1041031\_contig\_number\_26672
1
species
1871003

genus
3
239759


SRS098717\_contig\_number\_20241
1
species
1118061


SRS014948\_contig\_number\_18981SRS1055022\_contig\_number\_1475
species
2
28117
